# Short-term response to anti-VEGF as indicator of visual prognosis in refractory age-related macular degeneration

**DOI:** 10.1038/s41433-023-02900-6

**Published:** 2024-01-26

**Authors:** Anthony Gigon, Antonio Iskandar, Sophie Kasser, Sacha Naso, Marta Zola, Irmela Mantel

**Affiliations:** Department of Ophthalmology, University of Lausanne, Jules Gonin Eye Hospital, Foundation Asile des Aveugles, Lausanne, Switzerland

**Keywords:** Prognostic markers, Macular degeneration

## Abstract

**Background:**

Some patients with neovascular age-related macular degeneration (nAMD) respond insufficiently to anti-VEGF treatment despite maximal monthly intravitreal injections. Their short-term response between injections was investigated for extent and visual prognosis.

**Subjects/Methods:**

Monocentric retrospective observational study. 45 eyes from 41 patients with refractory nAMD (who previously received at least 12 months of anti-VEGF treatment), evaluated by optical coherence tomography (OCT) in between monthly anti-VEGF injections. The fluid profile on OCT was evaluated before, 1 week after, and 1 month after an intravitreal injection, using central retinal thickness (CRT), manual measurements, and fluid specific volumetric measurements performed by an automated algorithm based on artificial intelligence.

**Results:**

A significant improvement was found at week 1 in terms of CRT (*p* < 0.0001), intraretinal (IRF) (*p* = 0.007), subretinal fluid (SRF) (*p* < 0.0001), and pigment epithelium detachment (PED) volume (*p* < 0.0001). Volumetric fluid measures revealed a >50% reduction at week 1 for both IRF and SRF for approximately two-thirds of eyes. Poorer short-term response was associated with larger exudative fluid amounts (IRF + SRF) (*p* = 0.003), larger PED (*p* = 0.007), lower visual acuity (*p* = 0.004) and less anatomic changes at treatment initiation (*p* < 0.0001). Univariate and multivariate analysis revealed that visual outcomes 4 and 5 years later was significantly worse with weaker short-term responsiveness (*p* = 0.005), with the presence of atrophy (*p* = 0.01) and larger PED volumes (*p* = 0.002).

**Conclusions:**

Incomplete responders to anti-VEGF showed a significant short-term response, identifiable at 1 week after injection, with rapid recurrence at 1 month. Weaker short-term responsiveness at 1 week was associated with poorer long term visual prognosis. These patients may need adjuvant treatment to improve their prognosis.

## Introduction

Since the advent of anti-VEGF treatment, the visual prognosis of patients with neovascular age-related macular degeneration (nAMD) has changed dramatically [1–3]. These good results can be achieved at the cost of regular and timely treatment. However, some eyes remain incomplete responders in that exudative fluid may persist despite maximal monthly treatment. These cases have been referred to as refractory patients, incomplete responders, or resistant to anti-VEGF [4, 5]. Fluid dynamics following an anti-VEGF intravitreal injection in incomplete responders have been studied [6, 7]. While most cases show a good short-term response with quick relapse, a proportion of patients show persistent fluid that is truly refractory [7].

Although untreated or undertreated exudative activity is a reason for eventual visual acuity (VA) loss in nAMD, the visual prognosis of incomplete responders despite maximal treatment is less clear. Some studies have reported relatively good retention of VA in these patients [8, 9]. Furthermore, recently several studies investigated whether some subretinal fluid (SRF) could be actively tolerated without visual loss [10–13].

We hypothesized that the short-term response to anti-VEGF as identified by fluid measurements in between injections may be a relevant factor for visual prognosis. Thus, the goal of the present study was to investigate the fluid dynamics in incomplete responders to anti-VEGF, with respect to the VA prognosis. In addition, we aimed to pinpoint associated clinical or imaging factors as a mean to better understand the pathogenesis.

## Methods

This study was performed as a retrospective chart and optical coherence tomography (OCT) imaging review, in the medical retina department of the University Eye Hospital Jules Gonin, Lausanne, Switzerland. The study was approved by the Swiss Federal Department of Health for retrospective data analysis (Commission cantonale d’éthique CER-VD protocol number 2017-02175) and was performed in accordance with the ethical standards of the Declaration of Helsinki. The need for written informed consent was waived by the ethics committee.

We identified a consecutive series of eyes with nAMD which underwent an intermediate visit at 1 week after injection due to their incomplete response to anti-VEGF, defined as presence of intraretinal fluid (IRF) and/or SRF at each monitoring visit associated with the injection dates, and despite monthly anti-VEGF injections. This approach constituted the routine clinical practice for patients in whom incomplete response was found for 6 months or more, after a minimum of 12 months of anti-VEGF treatment. In addition, the eye had to be on the same anti-VEGF agent (ranibizumab or aflibercept) for at least 6 months. Before this time frame, switching between anti-VEGF drugs was allowed to the investigators’ discretion. The period screened for these inclusion criteria was from November 2014 to January 2017.

Exclusion criteria were insufficient quality of spectral domain (SD)-OCT images, any adjuvant treatment during the preceding year, any confounding retinopathy, ongoing topical treatment with prostaglandins, and any intraocular surgery during the preceding 6 months.

The routine clinical treatment attitude was a no tolerance strategy for fluid, translating into ongoing monthly treatment as long as fluid remained present. In case of successful fluid suppression, treatment intervals were prolonged in 2 weekly steps.

The routine ophthalmic visit included medical and ophthalmic history, best corrected VA (BCVA) on an Early Treatment Diabetic Retinopathy Study (ETDRS) chart, intraocular pressure, and slit-lamp and dilated fundus examination. Patients underwent an SD-OCT examination on the Heidelberg Spectralis OCT (macular cube 6 mm, 49 lines; Heidelberg Engineering, Heidelberg, Germany) at each visit. For the present study, the date of the intermediate visit 1 week after an anti-VEGF injection determined the previous visit just before the anti-VEGF injection as study-baseline.

This baseline visit, the visit 1 week after the injection, and the following visit at 1 month post-injection were analyzed and the following parameters collected: age, sex, eye, duration of previous anti-VEGF treatment, current anti-VEGF agent used, BCVA (routinely measured on ETDRS chart), date of the preceding injection, central retinal thickness (CRT), the maximal distance from internal limiting membrane to the retinal pigment epithelium (RPE) on SD-OCT, and the maximum elevation of RPE from Bruch’s membrane. The A-scan location of the latter two parameters was determined on baseline images and measured on identical A-scans using the follow-up mode for the succeeding two timepoints. In addition, the OCT images from baseline and 1 week later were qualitatively compared by the same investigator (SK, blinded to the algorithmic evaluation) and assigned to one of the following response categories: good, defined as demonstrating IRF and SRF absorption of more than half the baseline volume; moderate, defined as demonstrating visible fluid reduction but not more than 50%, and poor, defined as demonstrating no clear change in fluid amounts (less than 10%). Additional information extracted from baseline OCT included the presence or absence of vitreomacular adherence or traction and the thickness of the subfoveal choroid measured on enhanced depth imaging. The same OCT images were exported from the Spectralis device and analyzed by an automated algorithm (using a convolutional neural network and reinforced by layer segmentation), developed by the group RetinAI for identification and quantification of IRF and SRF, as well as the RPE elevation from Bruch’s membrane. The details of the algorithm are published elsewhere [14]. In brief, the algorithm was tested in a specific set of OCT volumes and showed a satisfying performance for all three compartments. In particular, the volumetric correlation between human expert and algorithm measurements were high (correlation coefficient of 0.99 for both IRF and SRF, and 0.91 for the sub-RPE space.^9^ This algorithm performed the volumetric measurements of fluid and RPE elevation in this study.

The presence or absence of atrophy was determined on fundus autofluorescence imaging at study baseline, in doubtful cases in combination with OCT. The presence or absence of fibrosis was determined on fundus color imaging.

Furthermore, the functional and structural response after treatment initiation in the past was evaluated, one month after three monthly loading doses (best available visual acuity before treatment and after loading dose, CRT before and after loading dose, presence or absence of fluid after loading dose).

Angiography was routinely performed when an incomplete response to treatment was found, including fluorescein and indocyanine green angiography (ICGA) on the Heidelberg Retinograph (Heidelberg Engineering). Information extracted from the last available fluorescein and ICGA included the neovascularization type (occult/type 1, classic/type 2, or retinal angiomatous proliferation/type 3), presence of aneurysmal choroidal changes (polypoidal vasculopathy), presence and diameter of a choroidal feeder vessel on early ICGA frames, and presence of any inflammatory signs (disc hyperfluorescence and/or diffuse exudation from the RPE). The measured blood pressure was recorded for both systolic and diastolic values.

For statistical analysis, a Microsoft Excel 2010 spreadsheet (Microsoft, Redmond, WA) and JMP software for Windows (version 8.0.1, SAS Institute, Cary, NC) were used. Besides descriptive statistics, the t-test was used for analysis of paired parameters (changes over time). An association analysis was performed for two outcome measures: the proportion of fluid (IRF and SRF) remaining present at week 1 as compared to baseline, and the absolute volume of the same remaining fluid. To identify factors related to the outcome measures, we used logistic regression for categorical variables, and Pearson’s test for continuous variables. For factors with *p* ≤ 0.2 in the univariate analysis, a step-by-step multivariate analysis was performed to determine independent variables. For the statistical results, a two-tailed *p*-value of 0.05 or less was considered statistically significant.

## Results

A total of 45 eyes (24 right eyes, 21 left eyes) of 41 patients (29 females, 12 males, mean age 78 ± 6 years) were identified and included into the analysis. The number of injections preceding the study baseline was a mean of 27 ± 15 injections, distributed over a mean 32 ± 19 months. The mean interval between two injections within the study period was 31 ± 3 days just before baseline and 30 ± 4 days between baseline and the next injection. The anti-VEGF drug used during the 6 months preceding baseline and directly thereafter was ranibizumab in 22 eyes and aflibercept in 23 eyes.

At anti-VEGF treatment initiation, visual acuity improved after the three monthly loading doses from a mean 65.2 ± 14.8 to 71.4 ± 14.8 ETDRS letters, and central retinal thickness decreased form 396 ± 146 um to 308 ± 101 µm. However, after loading dose residual fluid was still present in 67% of the eyes (IRF and SRF in 16%, IRF only in 4%, SRF only in 47%).

The short term responsiveness results between monthly injections, this is one week after baseline, are summarized in Table 1. Structural outcome measures all showed a significant change one week after injection as compared to baseline. These significant changes were all lost at 1 month from the preceding injection (except the manual height measures which still suggested a reduction for the neuroretinal fluid + SRF (*p* = 0.0005), and for PED (*p* = 0.01)). The time interval from the preceding injections was not significantly different at the 1-month visit as compared to study baseline.

**Table 1:** Measurements from spectral domain optical coherence tomography for all study time points during the evaluated refractoriness to monthly anti-VEGF for neovascular age-related macular degeneration.

	Baseline	1 week after injection	*P* value	1 month after injection	*P* value
Clinical data					
Time interval from last injection (days ± SD)	31 ± 3	7 ± 1	<0.0001	30 ± 4	0.33
Mean corrected visual acuity Snellen (letters ETDRS ± SD)	20/35 (71 ± 13)	20/35 (71 ± 13)	0.28	20/34 (72 ± 14)	0.72
OCT data from spectralis					
CRT (µm ± SD)	442 ± 129	390 ± 129	<0.0001	433 ± 134	0.12
Maximum distance ILM – RPE (mean value in µm ± SD)	367 ± 61	300 ± 72	<0.0001	339 ± 79	0.0005
maximum elevation of RPE (mean value in µm ± SD)	262 ± 133	233 ± 134	<0.0001	254 ± 136	0.01
OCT data from AI algorithm					
Mean volume of intraretinal cystoid spaces (µm^3^ ± SD)	77 ± 167	29 ± 80	0.007	65 ± 153	0.10
Mean volume of subretinal fluid (µm^3^ ± SD)	173 ± 250	96 ± 203	<0.0001	203 ± 418	0.58
Mean volume of pigment epithelium detachment (µm^3^ ± SD)	989 ± 990	906 ± 948	<0.0001	976 ± 997	0.31

*SD* standard deviation, *CRT* central retinal thickness, *ILM* internal limiting membrane, *RPE* retinal pigment epithelium.

*p* value is calculated as paired *t*-test.

**Fig. 1:**
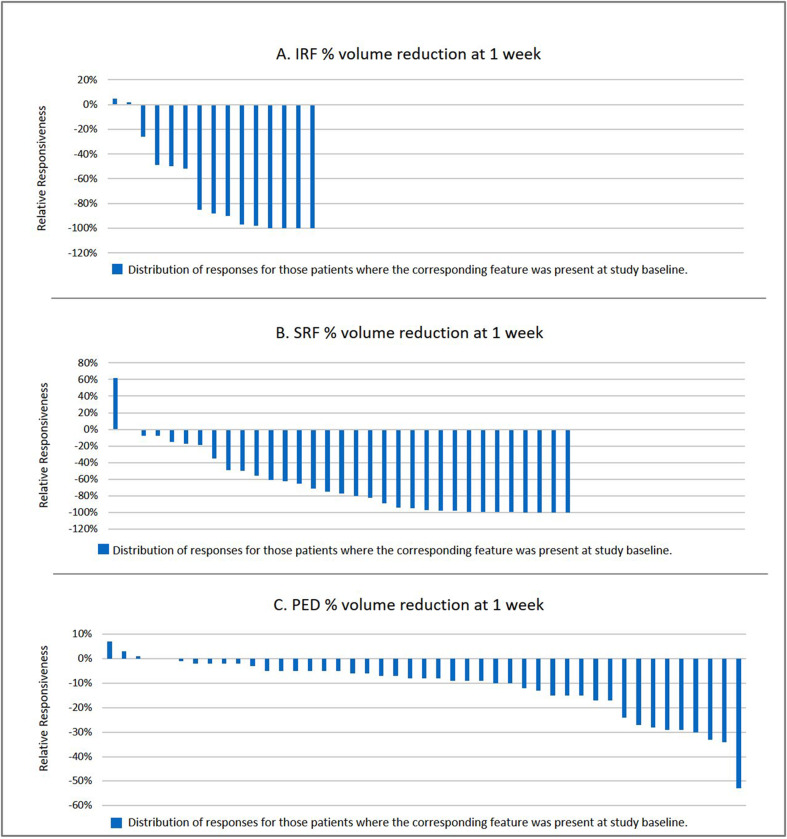
Distribution of the relative short-term change per fluid compartment. Each graph shows the distribution of relative responsiveness one week after intravitreal anti-VEGF injection per fluid compartment (**A** intraretinal fluid; **B** subretinal fluid; **C** retinal pigment epithelium detachment) in patients with neovascular age-related macular degeneration with incomplete response to anti-VEGF (intra- and/or subretinal fluid at 1 month after repetitive intravitreal anti-VEGF injection). Only eyes with a corresponding abnormal fluid compartment are shown. The relative short-term response 1 week after injection of anti-VEGF is calculated based on a fluid compartment measurement using a specifically developed and fully automated algorithm. **A** shows 15 study eyes with intraretinal fluid present at baseline (1 month after last injection) in the order of their percent of resolution 1 week after anti-VEGF injection as compared to baseline. The graph shows that most cases show more than 50% resolution of intraretinal fluid, but some eyes show no short-term response at all. **B** shows the short-term resolution distribution for subretinal fluid for the 33 eyes in which it was present at baseline. **C** shows the relative change distribution for the associated pigment epithelium detachment present in all study eyes.

Identifying eyes with the presence of IRF and SRF separately, defined as a volume of >10 µm^3^ at baseline, revealed 15 eyes with IRF (mean volume 222 ± 231 µm^3^) and 33 eyes with SRF (mean volume 238 ± 272 µm^3^), with 3 patients presenting both IRF and SRF. Both of these groups showed a mean reduction of fluid of 69% (range 0–100%). Figure 1A, B displays graphically the responsiveness between injections, grouped according to the subtype of fluid present, revealing that approximately two thirds of eyes with IRF or SRF respectively show 50% or more fluid reduction between injections. However, much less change was observed for PED, with only a 12% mean volume reduction at 1 week, although approximately one-quarter showed short-term volume changes between 20 and 55% (Fig. 1C).

As the definition of incomplete responders was based on the presence of IRF and/or SRF at each monthly visit, the sum of both was also analyzed (*n* = 45 eyes). The response distribution at 1 week revealed combined IRF + SRF volume reduction of ≥80%, ≥70%, ≥50%, ≥10%, in 49%, 58%, 76%, and 89% of eyes, respectively, However, in real-world settings, algorithmic fluid volume measures are not routinely available in clinics. Thus, we also categorized patients according to the clinical appreciation of OCT changes. This approach resulted in the following distribution: 44% were considered good responders (estimated more than 50% fluid resolution), 40% moderate responders (estimated ≤50% resolution but >10%), and 16% non-responders (estimated ≤10%). Examples can be found in the Supplementary Figure.

The responsiveness of exudative fluid (sum of IRF and SRF) was analyzed for its association with imaging factors. The distribution of imaging factors is listed in Table 2. The association with the short-term responsiveness of exudative fluid (IRF + SRF) was analyzed in univariate analysis. This revealed a significant negative correlation with baseline fluid volume of IRF + SRF (*r* = −0.43; *p* = 0.003), as well as PED (*r* = −0.40; *p* = 0.007). A weak, but statistically significant association was also found between baseline VA and short-term responsiveness (*r* = 0.09; *p* = 0.004), this is the better the vision the better the responsiveness. Finally, higher reduction in CRT (negative value) at initiation of anti-VEGF treatment was linked to less responsiveness of refractory fluid (*r* = 0.14; *p* < 0.0001). No other factor was significantly associated. Notably, there was no association with polypoidal vasculopathy or traction.

**Table 2:** Univariate factor analysis for factor associations with the responsiveness of refractory exudative fluid at 1 week after injection.

		Responsiveness of the refractory fluid (IRF + SRF)	
Continuous variables	Mean (SD)	Correlation (*p* value)	
Age	78.5 years (6.1)	*r* 0.22 (*p* = 0.15)	
Combined baseline volume of IRF + SRF	248 µm^3^ (276)	*r* −0.43 (***p*** = **0.003**)	
PED volume at baseline	989 µm^3^ (990)	*r* −0.40 (***p*** = **0.007**)	
Subfoveal choroidal thickness	170 µm (65)	*r* −0.11 (*p* = 0.48)	
Feeder vessel diameter on ICGA	129 µm (52)	*r* 0.04 (*p* = 0.79)	
Visual acuity in logMAR	0.24 (0.23)	*r* −0.09 (***p*** = **0.004**)	
CRT change at initiation of anti-VEGF treatment	−84 µm (148)	*r* 0.14 (***p*** < **0.0001**)	
Systolic blood pressure	140 mmHg (20)	*r* 0.05 (*p* = 0.72)	
Diastolic blood pressure	78 mmHg (11)	*r* 0.10 (*p* = 0.52)	
**Categorical variables**	***n***	**Mean (SD)**	**Anova** ***p*** **value**
Sex	Females 29 Males 12	0.66 (0.06) 0.67 (0.10)	*p* = 0.92
Macular neovascularization type	Type 1 = 36 Type 2 = 8	0.65 (0.06) 0.75 (0.11)	*p* = 0.40
PCV on ICGA	Present = 7 Absent = 38	0.66 (0.06) 0.74 (0.13)	*p* = 0.56
Inflammatory signs on fluorescein angiography	Present = 5 Absent = 40	0.66 (0.05) 0.74 (0.15)	*p* = 0.60
Vitreomacular adherence on OCT	Present = 7 Absent = 38	0.84 (0.13) 0.64 (0.05)	*p* = 0.14
Dryness achieved after initial loading dose	Yes 15 (33%) No 30 (67%)	0.68 (0.06) 0.77 (0.04)	*p* = 0.25
Fibrosis	Present = 4 Absent = 41	0.76 (0.12) 0.74 (0.04)	*p* = 0.89
Atrophy	Present = 6 Absent = 39	0.74 (0.10) 0.74 (0.04)	*p* = 0.98

*SD* standard deviation, *r* Pearson correlation coefficient, *IRF* intraretinal fluid, *SRF* subretinal fluid, *PED* pigment epithelium detachment, *CRT* central retinal thickness, *ICGA* indocyanine green angiography, *PCV* polypoidal choroidal vasculopathy.

**Fig. 2:**
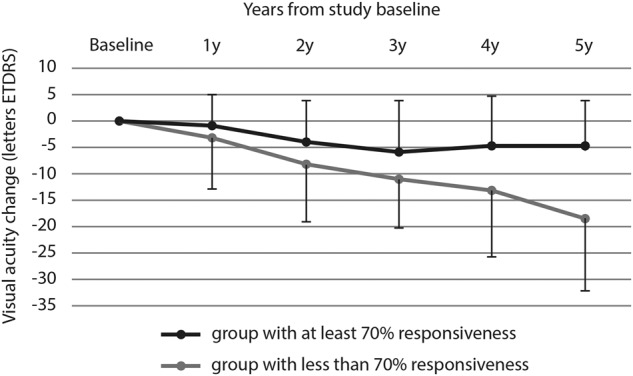
Visual acuity change (ETDRS letters) according to years of follow-up from study baseline. Eyes were subdivided into those with at least 70% responsiveness of the refractory exudative fluid (intraretinal + subretinal fluid) at 1 week from injection (*n* = 26), and those with less responsiveness (*n* = 19). The error bars indicate the standard deviation. The difference between the groups was significant after 5 years of follow-up (*p* = 0.016, analysis of variance).

Furthermore, the relevance of the responsiveness in terms of visual prognosis was investigated. Follow-up after the study period was available for an additional 1, 2, 3, 4, and 5 years for 42, 38, 35, 32, and 30 eyes, respectively. The available VA at yearly time points since evaluation of the short-term responsiveness showed a mean change of −2.0 ETDRS letters (standard deviation [SD] 8.0, *n* = 42), −5.4 letters (SD 9.9, *n* = 38), −7.3 (SD 10.0, *n* = 35), −7.3 (SD 12.1, *n* = 32), −8.9 (SD 12.8, *n* = 30), after 1, 2, 3, 4, and 5 years, respectively. The correlation analysis of the short-term responsiveness of exudative fluid with VA changes over the following 5 years revealed a significant correlation at years 4 and 5 (correlation factors 0.35 (*p* = 0.02) and 0.58 (*p* < 0.0001), respectively) (Table 3); the stronger the responsiveness between injections (in fluid percentage of IRF + SRF), the better the visual prognosis in the long run. The corresponding results after 1–3 years were not significant. Figure 2 shows the visual change over the follow-up duration from study baseline, according to the subgroups of at least 70% responsiveness of the refractory exudative fluid at 1 week after injection versus those with less responsiveness. While those with more than 70% responsiveness lost only one ETDRS line (5 letters) over 5 years, the less responsive group lost more than 3 ETDRS lines (>15 letters).

**Table 3:** Univariate and multivariate analysis for the association of the short-term refractoriness evaluation with long term visual outcomes (visual acuity changes) after additional 5 year follow-up.

Factor	Univariate analysis		Multivariate analysis
	*r* value	*P* value	*P* value
Relative responsiveness of IRF + SRF volume at 1 week after injection of anti-VEGF	0.58	<0.0001	0.005
IRF + SRF volume at study baseline (refractoriness just before anti-VEGF injection)	−0.30	0.045	n.s.
IRF + SRF volume at 1 week after injection of anti-VEGF	−0.31	0.037	n.s.
PED volume at study baseline	−0.51	0.0003	0.002
Presence of atrophy	n.a.	0.04	0.01
Presence of fibrosis	n.a.	0.27	
VA	0.36	<0.0001	n.s.
Initial responsiveness at treatment initiation	−0.01	0.67	

*r* Pearson correlation coefficient, *IRF* intraretinal fluid, *SRF* subretinal fluid, *VEGF* vascular endothelial growth factor, *n.s.* non-significant, *PED* pigment epithelium detachment, *n.a.* not applicable, *VA* visual acuity.

Exploring the correlation separately for those with IRF versus those with SRF confirmed this result for the group with SRF (*n* = 33) after 5 years of follow-up (*r* = 0.61, *p* = 0.0002). However, while the IRF group showed the same trend (*r* = 0.21) the group size with only 15 eyes was underpowered to be significant (*p* = 0.15).

The visual results after 5 years of follow-up showed significant correlations in univariate analysis with other factors as well (Table 3). A negative correlation was found for the volume of exudative fluid at study baseline (*r* = −0.30; *p* = 0.045), for residual fluid volume at week 1 (*r* = −0.31; *p* = 0.04), for baseline PED volume (*r* = −0.51; *p* = 0.0003), for presence of atrophy at baseline (*p* = 0.04). VA was also correlated with the loss of vision at year 5 (*r* = 0.36; *p* < 0.0001), meaning the better the VA, the more loss over 5 years. To determine their independent contribution, multivariate analysis with these significant factors was performed. The final model was statistically significant (*p* = 0.004), including the responsiveness of exudative fluid (*p* = 0.005), baseline PED volume (*p* = 0.002), and the presence of atrophy (*p* = 0.01) (Table 3).

## Discussion

Refractory nAMD, which could better be called incomplete responders to anti-VEGF, shows - according to this study – a significant response in between monthly anti-VEGF injections, at least in a large proportion (approximately two thirds) of cases. However, a smaller proportion of cases show no relevant short-term response. Associated risk factors were larger fluid amounts and larger PED, lower visual acuity and less central retinal thickness changes at treatment initiation. Furthermore, the study found that the degree of response between the monthly injections was relevant for long-term visual prognosis, identifiable after 4 and 5 years as greater visual loss in cases with poorer short-term response. These findings appear plausible: the more fluid and the longer it lasts, the worse the outcome. This was the case for the sum of IRF and SRF as well as for SRF alone, in spite of a very aggressive treatment attitude of no fluid tolerance. The numbers for IRF alone were too small for a meaningful conclusion. Additional factors for visual prognosis were the presence of atrophy and larger PED volumes. Initial VA was found to be correlated with the VA loss after 5 years, with more loss for better initial VA. This comes to no surprise because of the “ceiling effect” of anti-VEGF treatment: eyes with better VAs have less to gain and more to lose compared to eyes with poorer VAs.

Insufficient anti-VEGF response in nAMD with residual fluid despite maximal treatment is not a rare event, reported in up to 51% with fluid present (including IRF, SRF or PED) in the monthly ranibizumab treatment arm in the CATT study [15]. With respect to IRF and SRF alone, this proportion can be estimated as 48% and 34% for IRF and 33% and 25% for SRF according to monthly treatment arms with ranibizumab and aflibercept, respectively [16]. In our own prospective treatment series with ranibizumab, we found that approximately 16% of eyes still needed monthly treatment after 2 years of customized retreatment (observe-and-plan regimen), based on the presence of IRF or SRF [17].

So far, there is no consensus regarding the terminology and definition of cases with incomplete response to anti-VEGF. However, the presence of IRF and (recurrent) SRF on OCT has become a generally accepted indication for further treatment. Thus, we opted for this simple definition: that the presence of fluid despite maximal monthly treatment should be considered an incomplete response [4, 15]. The advantage of this definition goes along with an inhomogeneity in terms of short-term response in between injections, as shown in this study, and potentially with different pathophysiological backgrounds. Although a significant short-term response has previously been shown to occur already after 1 week as identified on CRT measurements [6], our recent study using a volumetric algorithm to determine precise fluid volumes showed important variability between cases in terms of the degree of response [7], consistent with the findings of the present study. To date, the short-term responsiveness in between injections has got little attention, and no clinical biomarkers in association have been described.

Visual function is the final purpose of eye healthcare. In a previous study, we reported surprisingly good visual outcomes over 3 years of (at least initially) refractory cases [8]. A slight disadvantage was seen if the refractory fluid included intraretinal cystoid spaces. However, these cases were investigated during their initial 3 years of treatment, contrasting with the present study which investigated cases during their follow-up treatment, according to the timepoint of short-term evaluation. They already had a mean preceding treatment period of 32 ± 19 months. Although the two studies are not directly comparable, it is intriguing that the different prognostic value of the short-term response between monthly injections in this study was seen late, after 4–5 years of further follow-up. Although quite late, this major difference is significant for patients’ lives, as the means differ by 15 letters (3 ETDRS lines) (Fig. 2). Thus, the visual consequences of fluid exudation are probably a product of several factors, including chronicity, quantity, location, and possibly its composition, inducing slow degenerative changes, leading ultimately to visual loss. However, long-term studies are not often available, with most studies being limited to 2 years [10, 13, 18]. We found only one small report with results at 5 years, suggesting good visual outcome despite subretinal fluid in 9 cases [9]. Not surprisingly, presence of atrophy and PED volume were also linked to worse visual prognosis at 4 and 5 years.

In addition to the evidence of the more deleterious effect of chronic non-responsive fluid, the dynamic curve of fluid amounts in between monthly anti-VEGF injections might reflect different pathogenic components of the fluid. The responsive part is visibly VEGF-dependent, while the residual amount of fluid which never disappears might be of different origin, being truly anti-VEGF refractory in its more restricted sense. We recently described a higher concentration of inflammatory biomarkers in aqueous humor in those with incomplete anti-VEGF response, as compared with normal responders [5]. In this context, it is interesting to consider the genetic background of AMD, which strongly implies the complement cascade [19].

Recently, a broad discussion about the importance of IRF versus SRF has suggested that persistent SRF might not be as harmful as IRF. This is based on the observation that mean VA is lower in the presence of IRF than in the presence of SRF, both at baseline and follow-up [3, 11, 15]. However, the comparisons do not consider the chronicity of presence of fluid but only their presence or absence at individual time points. The prospective FLUID trial was performed, allowing to actively tolerate up to 200 µm SRF if not responsive, and to extend treatment intervals in these cases [10]. With amazingly little effect on mean injection numbers, there was no significant visual difference between the relaxed and intensive (no tolerance) treatment arms, suggesting that SRF could be tolerated. However, a more recent post-hoc analysis of the same study showed a negative correlation between SRF volumes and visual outcomes [12]. Indeed, the precision of fluid volumetrics may help identify the visual consequences earlier in the time course.

Finally, fluid fluctuations have been reported to be more deleterious to visual function than small but stable amounts of fluid [20]. This may be linked to relative undertreatment. Our report seemingly contradicts this, although the study type differs significantly. While the reports of deleterious fluid fluctuation were based on recurrences under reduced treatment frequency, therefore possibly under-treatment, our study compared the treatment fluctuations under maximal monthly treatment. The lower treatment effect in our study shows as anti-VEGF resistant fluid which chronically stagnates (less fluctuations), ultimately leading to visual loss. Therefore, it may well be that both the completely non-responsive part of fluid and the intermittently undertreated but otherwise anti-VEGF-sensitive fluid might have similar negative effects in the long run.

A few weaknesses of this study need to be acknowledged. Besides the inherent weaknesses of a retrospective study and the limited size of the study sample, the clinical decision might have influenced the selection of patients in whom an intermediate visit at 1 week was performed, thus possibly influencing the selection of eyes for inclusion in this study. Furthermore, no subgroup analysis was performed to account for potential difference due to drug type (ranibizumab or aflibercept). Indeed, as the drug could be switched at the treating physician’s discretion, it would have been difficult, if not impossible to create subgroups according to the anti-VEGF molecule. However, as the difference between the duration of effect of both drugs is mild [21], it appears unlikely that this would impact the study results. In addition, the numbers of patients with intraretinal fluid did not allow a meaningful separate analysis of this subgroup.

In conclusion, while most incomplete responders to anti-VEGF showed a good short-term response to treatment, the evaluation of the short-term response between monthly injections may be of prognostic value: the more IRF and SRF is present (under the curve) over the month between injections, the poorer the visual prognosis over the following 5 years. Major fluid volumes with poor short-term response might need adjuvant treatment. However, further studies are needed to determine the nature of an adequate adjuvant treatment.

## Summary

### What was known before

- Neovascular age-related macular degeneration may be considered refractory in case of intra-/subretinal fluid present despite monthly intravitreal anti-VEGF treatment.

### What this study adds

- Approximately two thirds of cases showed good short term fluid reduction (more than 50%).
- However, around one third of cases showed little change of fluid in between injections. This was more likely in case of larger baseline fluid amounts, larger baseline PED, presence of polypoidal choroidal vasculopathy, or vitreomacular adherence.
- Short-term response was an indicator of visual prognosis, with low response correlating with poorer visual outcomes after 4 and 5 years of treatment.

### Supplementary information

Supplementary Figure

Supplementary Material

## Data Availability

The datasets used and/or analyzed during the current study are available from the corresponding author on reasonable request.
