# Supplementary figures and images for: Short-term response to anti-VEGF as indicator of visual prognosis in refractory age-related macular degeneration
Source: Eye (Lond). 2024 Jan 26;38(7):1342–8. doi: 10.1038/s41433-023-02900-6 (PMC11076480; doi:10.1038/s41433-023-02900-6)

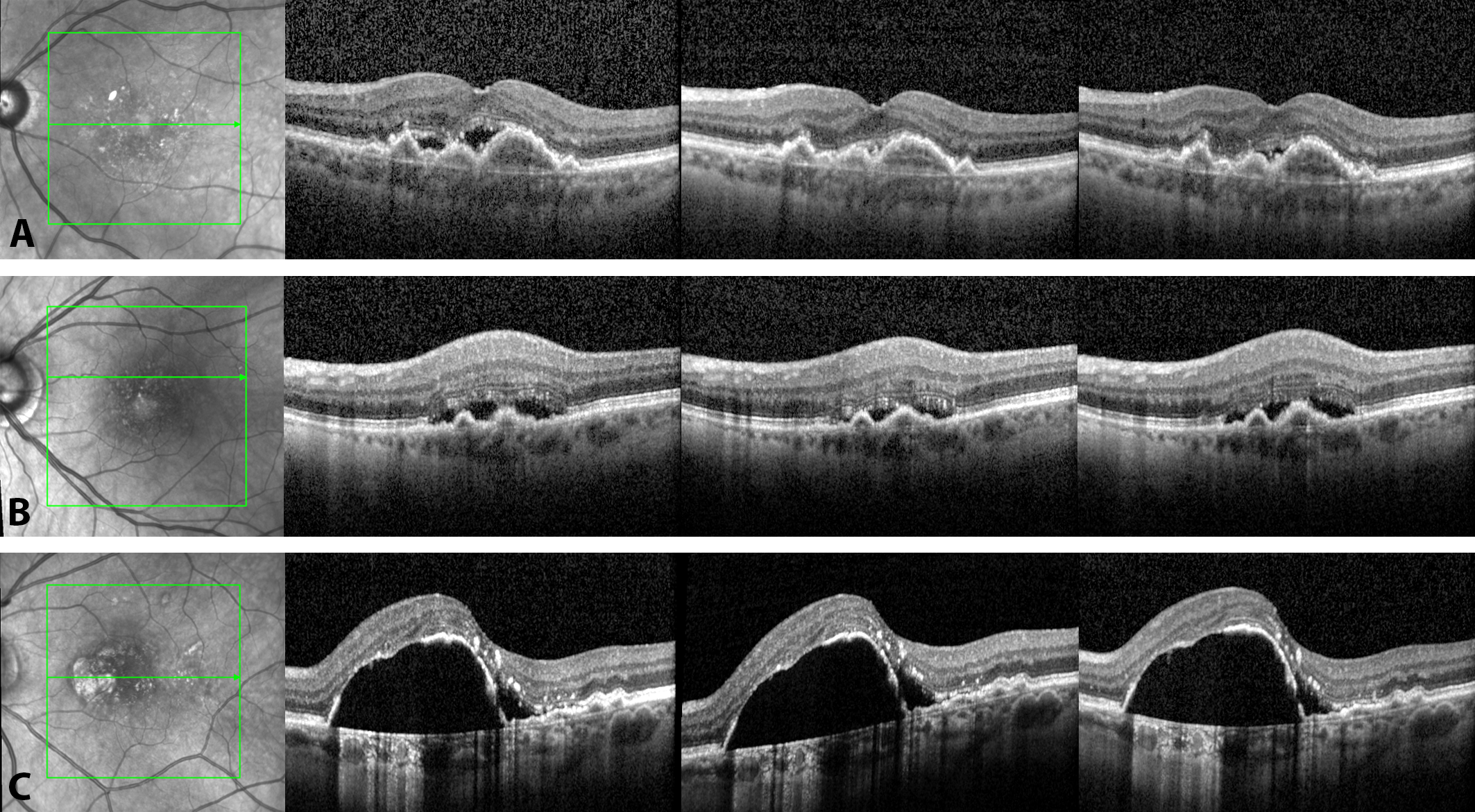

Supplement: Supplementary file 1 — Supplementary Figure [file 41433_2023_2900_MOESM1_ESM.tif]
