# Supplementary material for: Short-term response to anti-VEGF as indicator of visual prognosis in refractory age-related macular degeneration
Source: Eye (Lond). 2024 Jan 26;38(7):1342–8. doi: 10.1038/s41433-023-02900-6 (PMC11076480; doi:10.1038/s41433-023-02900-6)
Supplement: Supplementary file 2 — Supplementary Material [file 41433_2023_2900_MOESM2_ESM.docx]

**Supplementary Material Legend**

**Supplementary Figure. Examples of the response categories.**

The left column displays the B-scan location for all timepoints. The middle left shows baseline, middle right at 1 week post anti-VEGF injection, and the right column at 1 month after. A: Good responder (>50% fluid resorption at 1 week). B: Moderate responder (10-50% fluid resorption at 1 week). C: Poor responder (0–10% fluid resorption at 1 week).
